# Recurrent aggressive fibromatosis of the chest wall

**DOI:** 10.3332/ecancer.2014.464

**Published:** 2014-09-16

**Authors:** Riccardo Foà, Stefania Rizzo, Francesco Petrella, Federica De Maria, Massimo Bellomi

**Affiliations:** 1 Department of Health Sciences, University of Milan, via A. di Rudinì 8, Milan 20142, Italy; 2 Department of Radiology, European Institute of Oncology, via Ripamonti 435, Milan 20141, Italy; 3 Department of Thoracic Surgery, European Institute of Oncology, via Ripamonti 435, Milan 20141, Italy

**Keywords:** recurrent fibromatosis, chest, treatments

## Abstract

A 57-year-old woman with a previous history of aesthetic surgery for breast reduction presented with a subcutaneous mass in the right axilla. A CT scan showed a solid mass on the chest wall, and she underwent surgical resection with a diagnosis of aggressive fibromatosis. After a 10-month period of follow-up, a local recurrence occurred, and in accordance with the up-to-date approach, the recurrence has been treated with a conservative approach (medical treatments) with good control of the symptoms and downsizing of the lesion.

## Introduction

Aggressive fibromatosis (AF), also known as desmoid tumour or musculoaponeurotic fibromatosis, is a monoclonal fibroblastic proliferative disease [1]. The tumours are composed of spindle-shaped cells placed in a collagenous matrix without alterations, which is typical of malignancy. AF grows slowly and never metastasises; however, local recurrence and adjacent organ involvement are important causes of morbidity and mortality.

Available treatments include surgery, radiotherapy, systemic medical treatment, hormone therapy, anti-inflammatory drugs, and tyrosine kinase inhibitors. Recently, it has been demonstrated that an initial aggressive approach (surgery and/or radiotherapy) might overtreat 50% of the patients [2].

## Case

**Figure 1.**
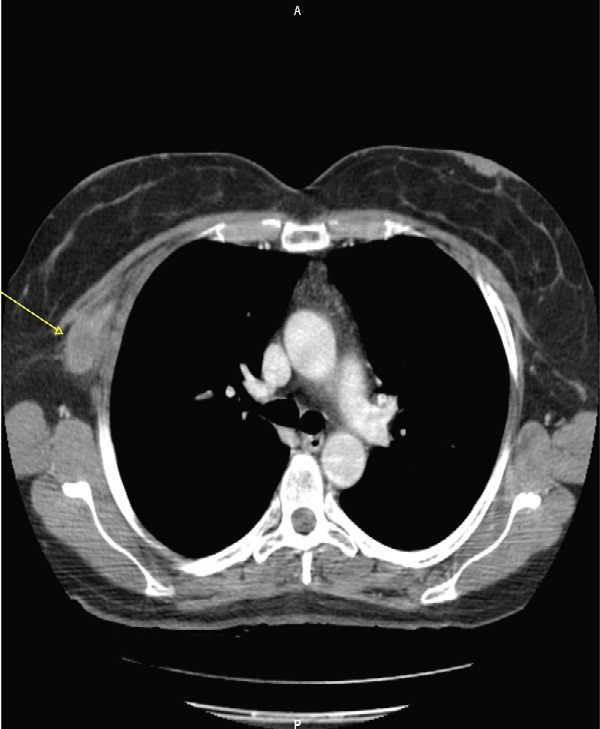
The axial CT scan at first diagnosis, showing a contrast-enhanced solid mass (arrow) in the right axilla, with a total extrapleural growth.

**Figure 2.**
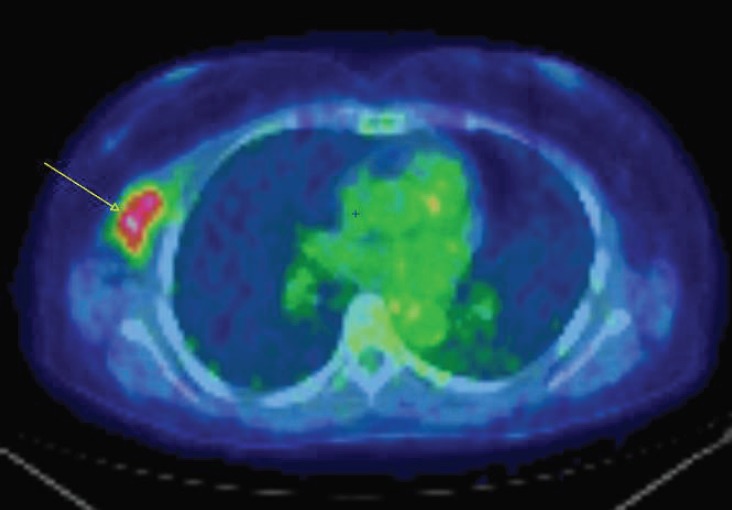
An axial fused PET-CT image shows an intense FDG uptake of the same lesion showed in Figure 1.

A 57-year-old woman with a previous history of aesthetic surgery for breast reduction presented with a subcutaneous mass in the right axilla. The patient underwent a computed tomography (CT) scan, showing a contrast-enhanced solid mass in the right axilla, with a total extra-pleural growth (Figure 1). A positron emission tomography (PET)-CT scan (Figure 2), showed an intense Fludeoxyglucose (FDG) uptake of the lesion, and a subsequent ultrasound (US)-guided biopsy disclosed mesenchymal tumour with uncertain malignant potential.

The patient underwent a wide soft-tissue surgical resection including major and minor pectoralis muscles, partial resection of the serratus anterior, and of the right breast upper external quadrant. The macroscopic pathological report described a white mass with a hard consistency and irregular margins, and the final histological diagnosis was extra-abdominal AF, with free resection margins, and an immune-phenotype positive for beta-catenin.

**Figure 3.**
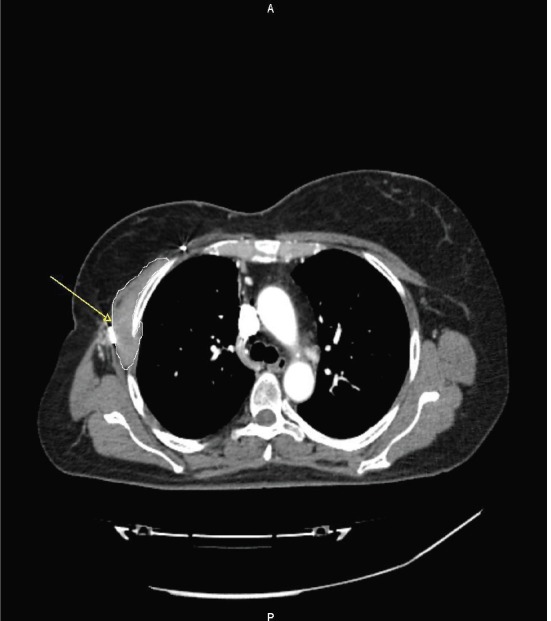
An axial CT scan at recurrence, showing a contrast-enhanced solid tissue (arrow) along the right chest wall, infiltrating the intercostal muscles and the pectoralis muscles, demonstrated at biopsy as recurrence of AF.

**Figure 4.**
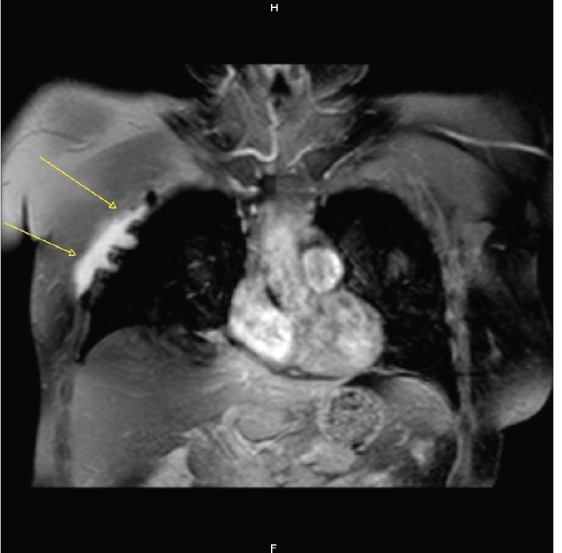
Coronal post-Gd MR T1 weighted image with fat suppression, showing the presence of an irregular enhancing solid tissue along the right chest wall (arrows), infiltrating the adjacent pleura and lung.

A follow-up CT scan performed 10 months after surgery disclosed local recurrence infiltrating the intercostal muscles, the pleura, and the pectoralis muscles (Figure 3). Subsequent MRI (Figure 4) demonstrated a 10 x 3.5 cm recurrence of AF, confirmed by the US-guided biopsy.

**Figure 5.**
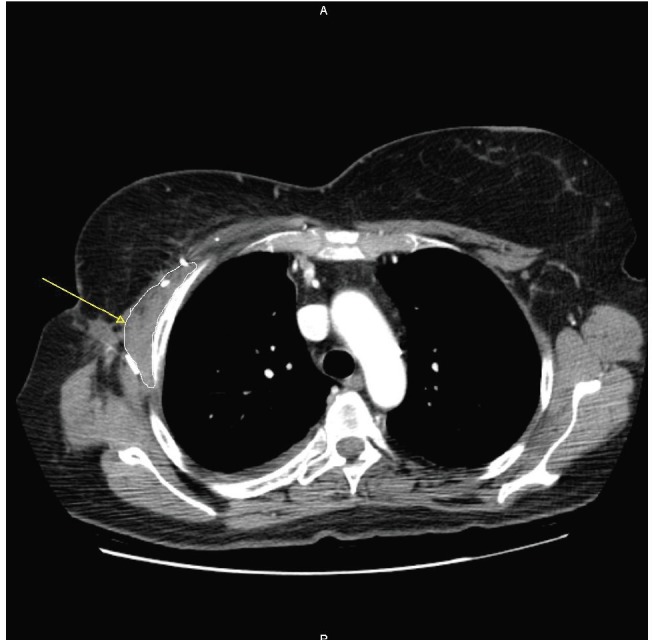
An axial CT scan, after 12 months of treatment, showing an initial downsizing of the lesion, contoured by a white line.

Based on the new strategies of approach, a treatment based on the use of tamoxifen and anti-inflammatory drugs, along with a gastric protection, was indicated for the patient. The therapy prescribed included tamoxifen 20 mg/diem and celecoxib 200 mg/diem (anti-inflammatory COX-2 inhibitor), with good control of the symptoms and (initial) downsizing of the lesion after 12 months of treatment, as demonstrated by CT scan (Figure 5).

## Discussion

AF (or desmoid tumour) is an uncommon mesenchymal tumour with a fibrotic band-like consistency, characterised by excessive proliferation of fibroblast-like cells [2].

The incidence of fibromatosis in the general population is 2–4 cases/million per year, with a slight female preponderance and peak incidences in the 3rd-4th decades of life [2]. AF is a locally aggressive tumour with no tendency to metastasise. However, local recurrence and adjacent organ involvement are important causes of morbidity and mortality.

The causes of AF are still unclear but have been related to trauma, hormonal factors, and genetic associations [such as adenomatous polyposis and mutations of the adenomatous polyposis coli (APC) gene] [3].

AF can be intra-abdominal or extra-abdominal, being located in the chest wall 8–10% of all the extra-abdominal sites [4].

Before 2000, radical surgical resection was considered to be the standard treatment because complete excision with negative margins was considered to be the primary goal, such as indicated for sarcomas.

However, nowadays, amputation is no longer considered the most appropriate treatment in locally advanced AF, because the consequences of radical excision can be worse than the disease itself. Moreover, the fact that some tumours recur after surgery, but then remain stable without treatment, suggests that growth factors released following surgery may promote recurrence in tumours that would be otherwise indolent.

A recently proposed treatment algorithm for AF [2] begins with a careful clinical assessment and a two-month follow-up, in order to discriminate between rapidly enlarging and indolent tumours, so that more aggressive therapies may be reserved for those who really need it.

Valid current alternative treatments to surgery and radiotherapy are the anti-oestrogen and anthracycline-containing regimen [5] of tamoxifen, anti-inflammatory drugs (such as sulindac, celecoxib, and indomethacin), tyrosine kinase inhibitors (such as imatinib [6]), and cryoablation [2].

In our case, surgical resection was performed because of unclear histology at biopsy; at the time of local recurrence, re-do surgery was avoided, and according to the reported advances in knowledge of therapies for AF, the patient has been treated with tamoxifen and celecoxib. We saw initial promising results with control of pain and partial reduction of the lesion after 12 months of treatment.

## Conclusion

In conclusion, the current treatment for AF should include medical treatments alternative to surgery and radiotherapy, as they are able to ensure good control of symptoms and reduce the possibility of postoperative complications.

## Conflicts of interest

The authors have no conflicts of interest to declare.
